# The anaphase-promoting complex/cyclosome: a new promising target in diffuse large B-cell lymphoma and mantle cell lymphoma

**DOI:** 10.1038/s41416-019-0471-0

**Published:** 2019-05-15

**Authors:** Anke Maes, Ken Maes, Hendrik De Raeve, Eva De Smedt, Philip Vlummens, Vanessa Szablewski, Julie Devin, Sylvia Faict, Kim De Veirman, Eline Menu, Fritz Offner, Marcel Spaargaren, Jérôme Moreaux, Karin Vanderkerken, Els Van Valckenborgh, Elke De Bruyne

**Affiliations:** 1 Department of Hematology and Immunology, Myeloma Center Brussels, Vrije Universiteit Brussel, Brussels, Belgium; 2 Department of Pathology, UZ Brussel, Vrije Universiteit Brussel, Brussels, Belgium; 3 Hematology, Department of Internal Medicine, Ghent University Hospital, Ghent, Belgium; 4 Department of Biopathology, CHU Montpellier, Montpellier, France; 5 Laboratory for Monitoring Innovative Therapies, Institute of Human Genetics, CNRS, Montpellier, France; 6 Department of Pathology, Lymphoma and Myeloma Center Amsterdam (LYMMCARE), Cancer Center Amsterdam, Amsterdam UMC, University of Amsterdam, Amsterdam, the Netherlands

**Keywords:** B-cell lymphoma, Mitosis

## Abstract

**Background:**

The aggressive B-cell non-Hodgkin lymphomas diffuse large B-cell lymphoma (DLBCL) and mantle cell lymphoma (MCL) are characterised by a high proliferation rate. The anaphase-promoting complex/cyclosome (APC/C) and its co-activators Cdc20 and Cdh1 represent an important checkpoint in mitosis. Here, the role of the APC/C and its co-activators is examined in DLBCL and MCL.

**Methods:**

The expression and prognostic value of Cdc20 and Cdh1 was investigated using GEP data and immunohistochemistry. Moreover, the therapeutic potential of APC/C targeting was evaluated using the small-molecule inhibitor proTAME and the underlying mechanisms of action were investigated by western blot.

**Results:**

We demonstrated that Cdc20 is highly expressed in DLBCL and aggressive MCL, correlating with a poor prognosis in DLBCL. ProTAME induced a prolonged metaphase, resulting in accumulation of the APC/C-Cdc20 substrate cyclin B1, inactivation/degradation of Bcl-2 and Bcl-xL and caspase-dependent apoptosis. In addition, proTAME strongly enhanced the anti-lymphoma effect of the clinically relevant agents doxorubicin and venetoclax.

**Conclusion:**

We identified for the first time APC/C as a new, promising target in DLBCL and MCL. Moreover, we provide evidence that Cdc20 might be a novel, independent prognostic factor in DLBCL and MCL.

## Background

Diffuse large B-cell lymphoma (DLBCL) and mantle cell lymphoma (MCL) are among the most common aggressive B-cell non-Hodgkin lymphomas (NHL). Although the standard-of-care regimen R-CHOP (rituximab, cyclophosphamide, doxorubicin, vincristine and prednisone) has significantly improved the survival rates, MCL remains incurable and up to one-third of the DLBCL patients are or become refractory.^1,2^ Within the DLBCL patients, two major DLBCL subtypes are identified by gene expression profiling: germinal centre B cell (GCB) and activated B-cell (ABC) DLBCL, of which the ABC–DLBCL subtype has a worse clinical outcome.^3^

The clinical use of cell cycle targeting agents, such as the microtubule-targeting agent vincristine, has proven that this is an interesting approach in the treatment of high-grade B-cell NHL.^4^ However, these agents induce severe toxicity and are associated with multidrug resistance development.^5^ Clinical studies are currently ongoing to find more selective cell cycle targets by blocking either the interphase (e.g. Cdk inhibitors) or mitotic entry (e.g. microtubule-targeting agents, Plk-1 and aurora kinase inhibitors).^6^ In B-cell NHL, the anti-lymphoma effects of these agents were either disappointing or the trials were suspended early due to toxicity issues.^6–9^ Recent preclinical studies show that targeting molecules involved in the mitotic exit, such as the anaphase-promoting complex/cyclosome (APC/C), is a better strategy since it provokes a more permanent mitotic arrest, thereby reducing the chance of mitotic slippage and thus enhancing mitotic cell death.^10^ The activation of the APC/C, an E3-ubiquitin ligase, depends on the interaction with its co-activators Cdc20 or Cdh1. The spindle assembly checkpoint (SAC) prevents APC/C activation in early mitosis. When bi-orientation of the chromosomes is achieved in the metaphase, the SAC pathway is inactivated and APC/C–Cdc20 binding occurs. This interaction leads to proteasomal degradation of the substrates cyclin B1 and securin, subsequently allowing the onset of the anaphase.^11^ Cdc20 is thereafter replaced by Cdh1 and the APC/C–Cdh1 targets Cdc20, aurora kinases and Plk-1 during mitotic exit. In early G1 phase, Skp2 and mitotic cyclins are degraded, resulting in increased p21, p27 and cyclin D levels, which is necessary to maintain the G1 phase.^11,12^

Several studies have already suggested the involvement of the APC/C and its co-activators in tumorigenesis of different cancers and their potential as a new therapeutic target.^11,13^ Wang et al. were the first to demonstrate APC/C mutations in human cancer cells, showing that a disruption of some of the APC/C subunits contributes to tumorigenesis by deregulation of key cell cycle regulators.^14^ Increased Cdc20 expression is correlated with poor prognosis in several human cancers,^15–21^ and depletion of Cdc20 in various cancer cell lines resulted in tumour metaphase arrest and induction of apoptosis.^19,22,23^ Moreover, inhibition of Cdh1 has been associated with tumorigenesis and decreased Cdh1 expression is observed in breast and colon cancer.^24,25^ The small molecule TAME (tosyl-L-arginine methyl ester) was discovered as a specific APC/C inhibitor in the past decade. It mimics the IR-tail of the co-activators and blocks the interaction between APC/C and Cdc20 or Cdh1.^26^ Previously, we and others demonstrated that proTAME (permeable variant of TAME) treatment results in a reduced viability, a growth arrest and apoptosis of malignant plasma cells.^15,27^

Currently, however, little is known about the therapeutic potential of targeting the APC/C and its co-activators in DLBCL and MCL. The aim of this study is to investigate the expression of the APC/C co-activators Cdc20 and Cdh1 and the therapeutic potential of APC/C targeting in DLBCL and MCL.

## Methods

### Analysis of Cdc20 and Cdh1 gene expression levels

The publicly available GEP datasets GSE10846^28,29^ (containing gene expression data and survival data of 167 ABC–DLBCL patients, 183 GCB–DLBCL patients and 64 unclassified DLBCL patients), GSE56315^30^ (containing gene expression data of 33 B- cell samples, 23 ABC–DLBCL patients, 29 GCB–DLBCL patients and 3 unclassified DLBDL patients), GSE16455^31^ (containing gene expression data of 7 indolent and 15 aggressive MCL patients) and GSE36133^32^ (containing gene expression data of 13 human DLBCL cell lines and 5 MCL cell lines) were used. Raw CEL files were obtained from the Gene Expression Omnibus (GEO) and gcrma-normalisation was performed in R using bioconductor. Survival analysis of GEP microarray data was done using Genomicscape (http://genomicscape.com). The following probe sets were used: 202870-s-at (Cdc20) and 209416-s-at (Cdh1).

### Patient biopsies and staining

Patient samples were collected at the Department of Pathology in Brussels (UZ Brussels, Belgium) and the Department of Biopathology in Montpellier (CHU Montpellier, France). Paraffin-embedded samples available from seven DLBCL patients were selected in Brussels and 3-μm-thick sections from tissue microarrays containing three representative 0.6-mm cores of routinely processed tissues from 27 DLBCL patients were included from Montpellier.^33^ All samples were stained with Cdc20 antibody (ATLAS Antibodies AB, Bromma, Sweden) using an automated immunostainer Benchmark XT (Roche Ventana, Basel, Switzerland). As a positive control, tonsils with secondary follicles were used. Two hundred tumour cells were counted and the percentage of tumour cells with a staining of any intensity was determined at a magnification ×400 on a Leica DM2000 microscope. The counting was performed in hot-spot areas by means of an ocular grid. Based on the R-IPI of the DLBCL patients, they were divided into good and poor prognosis groups. Cells were scored MYC and Bcl-2 positive as we described previously.^33^

### Cell culture

All the MCL (Jeko-1, Mino and Rec-1) and ABC–DLBCL cell lines (U2932 and RI-1) were maintained in the RPMI-1640 medium (Lonza, Basel, Switzerland) supplemented with 10% foetal calf serum (FCS) (Biochrom AG, Berlin, Germany) and 2 mM glutamine (Life Technologies, Gent, Belgium). The GCB–DLBCL cell lines (SU-DHL-6, OCI-Ly1 and OCI-Ly7) were maintained in the IMDM medium (Life Technologies) supplemented with 10% FCS and 2 mM glutamine. Cells were cultured at 37 °C in a humidified 5% CO_2_atmosphere. All cell lines were obtained from ATCC and regularly tested for mycoplasma contamination. They were authenticated by STR profiling.

### Reagents

The APC/C inhibitor proTAME was obtained from R&D Systems (Oxon, UK). Venetoclax and rituximab were obtained from Selleckchem (Bio-Connect, Huissen, The Netherlands), doxorubicin hydrochlorate was purchased from Sigma-Aldrich (Bornem, Belgium) and apcin was kindly provided by Dr. R.W. King (Department of Cell Biology, Harvard Medical School).

### Statistical analysis

Prognostic significance of Cdc20 and Cdh1 gene expression was calculated using the MaxStat R package. Statistical differences in overall survival were calculated by a log-rank test and survival curves were plotted using Kaplan–Meier method. Multivariate analysis was performed using the Cox proportional hazards model. Graphical and statistical analysis was performed using GraphPad Prism 5.01 software. Statistical significance (*p*-value of *p* < 0.05 was considered significant) was determined by a Mann–Whitney U test (to compare two groups) and a one-way ANOVA with Bonferonni correction for multiple testing.

More details on the ‘Methods' section are described in Supplemental Methods.

## Results

### Cdc20 expression is increased in DLBCL and MCL patients and associated with poor survival

**Fig. 1:**
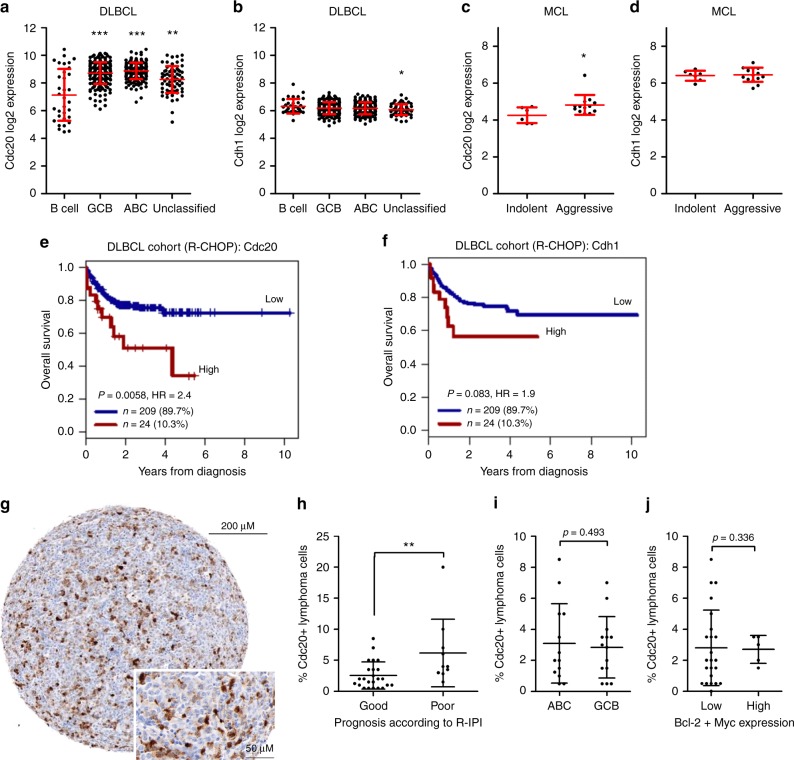
Cdc20 mRNA expression is increased in DLBCL and MCL patients and is associated with worse survival in DLBCL patients. **a**, **b** Cdc20 and Cdh1 mRNA expression in DLBCL. Cdc20 (**a**) and Cdh1 (**b**) gene expression levels of B-cell samples (*n* = 33), patients with ABC–DLBCL (*n* = 190), patients with GCB–DLBCL (*n* = 212) and patients with unclassified DLBCL (*n* = 67) were obtained from the publicly available microarray datasets GSE10846 and GSE56315. Mean expression±SD is shown in red. **p < 0.01 and ***p < 0.001. **c**, **d** Cdc20 and Cdh1 mRNA expression in indolent and aggressive MCL. Cdc20 (**c**) and Cdh1 (**d**) gene expression levels of patients with indolent (*n* = 7) and aggressive MCL (*n* = 15) were obtained from the GSE16455 dataset. Mean expression ± SD is shown in red. *p < 0.05. **e**, **f** Prognostic value of Cdc20 and Cdh1 mRNA levels in terms of overall survival in DLBCL. The prognostic value of Cdc20 (**e**) and Cdh1 (**f**) was determined in DLBCL patients receiving R-CHOP treatment from the Lenz cohort (*n* = 233) using Maxstat analysis (cut-off value used for Cdc20 is 11.679 and for Cdh1 8.319). Data were analysed through genomicscape (http://genomicscape.com). **g**–**j** Immunohistochemical analysis of Cdc20 expression in primary DLBCL patient samples. Immunohistochemical analysis of Cdc20 expression in a DLBCL patient. A ×4 and ×40 magnification is shown (**g**). Percentage of Cdc20-positive lymphoma cells was counted in 34 DLBCL patients and plotted against the estimated prognosis according to the R-IPI score of the patients (**h**). Percentage of Cdc20-positive cells was also plotted against the molecular subtype (**i**) and double-expresser status (**j**) in 27 DLBCL patients

To investigate the clinical relevance of the APC/C in the aggressive B-cell malignancies DLBCL and MCL, the gene expression levels of the two APC/C co-activators, namely Cdc20 and Cdh1, were analysed using publicly available gene expression profiling (GEP) datasets. The expression of Cdc20 and Cdh1 mRNA was assessed in DLBCL patients, using GEP data from B-cell samples (*n* = 33), ABC–DLBCL (*n* = 190), GCB–DLBCL (*n* = 212) and unclassified DLBCL patients (*n* = 67). The gene expression levels of Cdc20 were significantly increased in all DLBCL subtypes compared with the B-cell samples. In contrast, the Cdh1 gene expression levels were not significantly different in the GCB–DLBCL and ABC–DLBCL patients compared with the B-cell samples (Fig. 1a, b). Analysis of gene expression levels in a MCL cohort revealed elevated Cdc20 expression levels in aggressive MCL compared with indolent MCL. No significant difference was observed for Cdh1 expression (Fig. 1c, d). Importantly, high Cdc20 expression levels were associated with a worse survival in DLBCL patients receiving the standard R-CHOP treatment. The same trend was observed for Cdh1 and clinical outcome; however, significance was not reached (Fig. 1e, f).

Next, the prognostic value of Cdc20 expression in DLBCL patients was compared with conventional prognostic factors, including the age at diagnosis, International Prognostic Index (IPI) and molecular DLBCL subtypes. Univariate COX analysis demonstrated that all these factors have a prognostic value in DLBCL (Table 1A). In the multivariate COX analysis, when all parameters were tested together, Cdc20 expression, IPI and molecular subtype remained independent prognostic factors (Table 1B). Finally, the protein Cdc20 expression was determined on DLBCL patient biopsies (Fig. 1g). The percentage Cdc20-positive lymphoma cells was counted and plotted against the patient’s prognosis according to their R-IPI. A significant higher amount of Cdc20-positive lymphoma cells was counted in the patients with a poor prognosis compared with the patients with a good prognosis (*p* = 0.0042) (Fig. 1h). Consistent with the multivariate COX analysis, neither significant correlation between Cdc20 expression and molecular subtype was found (Fig. 1i; Table 1B) nor double-expresser status (Fig. 1j). Together, these data suggest that increased Cdc20 expression is associated with high-grade lymphoma and Cdc20 thus represents an interesting therapeutic target.

**Table 1:** COX univariate and multivariate analysis of overall survival in R-CHOP-treated DLBCL (*n* = 233) including Cdc20 gene expression

A. Univariate analysis	Overall survival (*n* = 233)	
Prognostic variable	HR	*p*-value
Cdc20	2.43	0.008
Age (>60 years)	2.2	<0.0001
GCB–ABC molecular subgroups	2.75	<0.0001
IPI	1.79	<0.0001

B. Multivariate analysis	Overall survival (*n* = 233)	
Prognostic variable	HR	*p*-value
Cdc20	2.91	0.01
Age (>60 years)	0.79	ns
GCB–ABC molecular subgroups	4.15	<0.0001
IPI	1.64	0.001

The prognostic factors were tested as a single variable (A) or multi variables (B) using the COX-model. *p*-values and hazard ratios (HR) are shown. ns: not significant at a 5% threshold

### Pharmacological inhibition of the APC/C decreases lymphoma cell viability and induces apoptosis

**Fig. 2:**
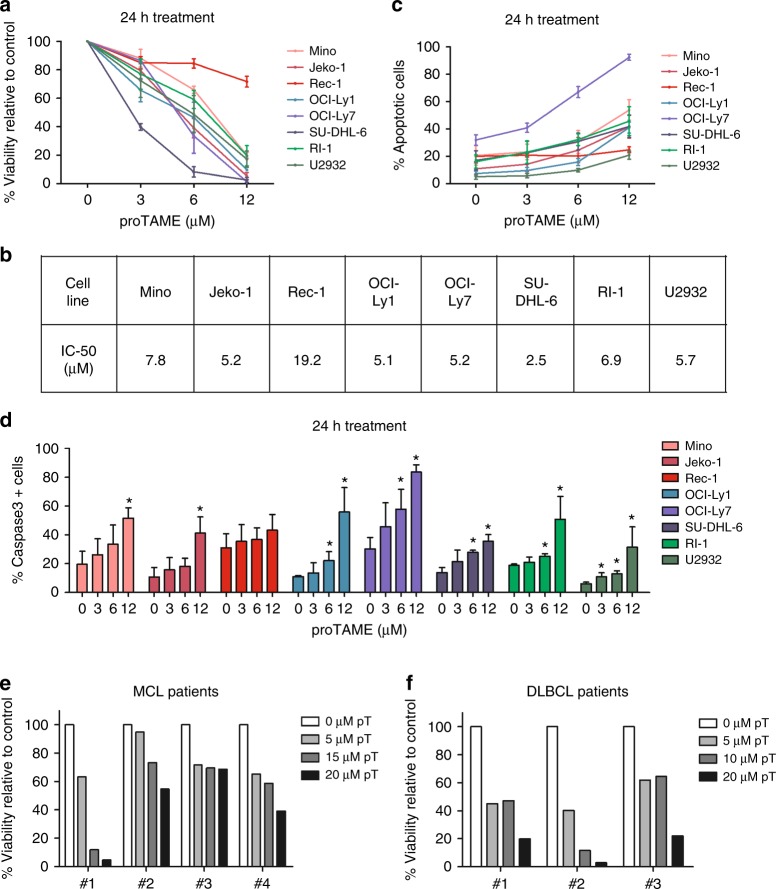
APC/C inhibition reduces DLBCL and MCL viability and induces caspase-3-mediated apoptosis. **a**, **b** Effect of proTAME treatment on cell viability. MCL and DLBCL cell lines were treated for 24 h with proTAME (3, 6 and 12 µM) and the effect on viability was determined using a CellTiter-Glo assay (**a**). The results are shown as % viability relative to control. The IC-50 values for each cell line were calculated using Prism (**b**). **c**, **d** Effect of proTAME treatment on apoptosis. The effect of proTAME treatment on apoptosis (**c**) and percentage of active caspase-3- positive cells (**d**) was determined after 24 h, using an Annexin V/7′-AAD staining, followed by flow-cytometric analysis, and active caspase-3 staining followed by flow-cytometric analysis. The percentage of apoptotic cells are the sum of the percentage of Annexin V and Annexin V/7′-AAD positive cells. The results shown in graphs **a**, **c** and **d** are the mean ± SD of three independent experiments. *p < 0.05, **p < 0.01 and ***p < 0.001. **e**, **f** The effect of proTAME treatment on primary patient samples. The viability of the proTAME-treated MCL (**e**) and DLBCL (**f**) patient samples was determined after 24 h. The results are shown as % viability relative to control

Next, the APC/C was investigated as a potential target in DLBCL and MCL using human cell lines. Therefore, the Cdc20 and Cdh1 expression was first determined using gene expression profiling data of a large panel of DLBCL and MCL cell lines. Similar to the primary patient samples, we found a significantly higher Cdc20 mRNA expression in both the DLBCL and MCL cell lines compared with B-cell samples and a lower Cdh1 mRNA expression (Supplemental Fig. 1a). These findings were also confirmed both at mRNA and protein levels in a selected panel of DLBCL and MCL cell lines (Supplemental Fig. 1b, c). Next, the effect of the specific APC/C inhibitor proTAME was evaluated on the viability of the selected MCL and DLBCL cell lines. As shown in Fig. 2a, a dose-dependent decrease in viability was observed in all cell lines after 24 h of treatment. Among the MCL cell lines, the Rec-1 cells were found the least sensitive to proTAME (IC-50 of 19.2 µM), while the Jeko-1 cells were the most sensitive with an IC-50 of ~5.2 µM. Most of the DLBCL cell lines have an IC-50 value around 6 µM, except for the SU-DHL-6 cells which were the most sensitive with an IC-50 of 2.5 µM (Fig. 2b). This decreased viability was also confirmed by performing cell counting (Supplemental Fig. 1d). The effect of APC/C inhibition was also determined on apoptosis by using an Annexin V/7′-AAD staining. A significant increase in apoptotic cells was observed in all cell lines (Fig. 2c). Moreover, this induced apoptosis seems to be caspase-3 mediated, as evidenced by a significant increase in the percentage of active caspase-3-positive cells after APC/C inhibition (Fig. 2d). Similar effects on viability and apoptosis were observed after 48 h of treatment (Supplemental Fig. 1e–h). Finally, the effect of pharmacological inhibition of the APC/C was tested on primary patient cells obtained from four MCL and three DLBCL patients. As shown in Fig. 2e, f, a dose-dependent reduction in viability was observed in all patient samples after 72 h of proTAME treatment. Patient characteristics can be found in Supplemental Table 1.

### APC/C inhibition results in a metaphase arrest

**Fig. 3:**
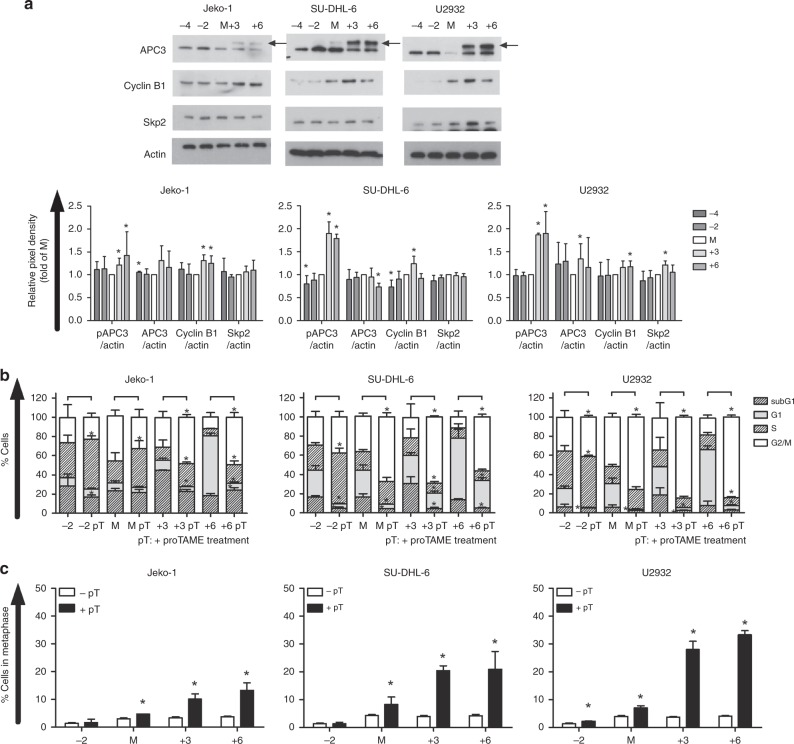
Pharmacological inhibition of the APC/C with proTAME results in a metaphase arrest. **a** Effect of proTAME treatment on the substrates of the APC/C co-activators Cdc20 and Cdh1. Expression of cyclin B1 and Skp2 was determined at different timepoints in the MCL and DLBCL cell lines after synchronisation and release into proTAME treatment (3 µM in SU-DHL-6 and 6 µM in Jeko-1 and U2932). APC3 and β-actin were used as indicators of mitotic arrest and loading control. Arrows show the phosphorylated APC3. One experiment representative of three is shown (the results shown are from the same experiment as those shown in Fig. 4). Normalisation was performed with Image J and quantification relative to the M condition is shown. Bars represent the mean± SD of three independent experiments. **b**, **c** Effect of proTAME treatment on cell cycle progression. The effect on cell cycle progression was determined using PI staining on cell lines treated with 3 µM (SU-DHL-6) or 6 µM (Jeko-1 and U2932) proTAME for the indicated timepoints after synchronisation (**b**). To further examine the effect on mitosis, cytospins of these treated cells were stained with May–Grünwald Giemsa. For each sample, 3 × 100 cells were counted per cytospin. Quantification of the percentage of cells in the metaphase is shown (**c**). The results shown in each graph are the mean ± SD of three independent experiments. *p < 0.05 and **p < 0.01. M = mitosis, −4 = 4 h before mitosis, −2 = 2 h before mitosis, +3 = 3 h after mitosis and +6 = 6 h after mitosis

To determine the underlying mechanisms of action, the effect of proTAME on the activity of the co-activators was assessed by determining the protein levels of the APC/C–Cdc20 substrate cyclin B1 and APC/C–Cdh1 substrate Skp2. Cells were synchronised using a double thymidine block and released into proTAME treatment. Western blot analysis demonstrated that cyclin B1 protein levels increased during mitotic arrest in all MCL and DLBCL human cell lines used, as indicated by phosphorylation of the APC/C subunit APC3 (also known as Cdc27).^34^ Skp2 protein levels remained mostly unchanged, except in the ABC–DLBCL cell line (Fig. 3a). In contrast, in untreated synchronised cells, neither significant accumulation of cyclin B1 or Skp2 levels was observed, nor was there an increase in phosphorylation of APC3 (Supplemental Fig. 2). Together, these data indicate that the induced mitotic arrest is proTAME-dependent, as well as the effects seen on cyclin B1 and Skp2 protein levels. As the APC/C is an important protein complex involved in the metaphase–anaphase transition during mitosis, the effect on cell cycle progression was investigated. The mitotic arrest was confirmed by cell cycle analysis using a propidium iodide flow-cytometry staining (Fig. 3b). To further validate these findings, a May–Grünwald Giemsa staining was performed and cells in the metaphase were quantified (Supplemental Fig. 3). A significant increase in the percentage of cells in the metaphase after APC/C inhibition was observed (Fig. 3c). These findings on synchronised cells demonstrate that proTAME treatment induced metaphase arrest in DLBCL and MCL cells.

### Induced apoptosis is mediated by phosphorylation of Bcl-2 and Bcl-xL

**Fig. 4:**
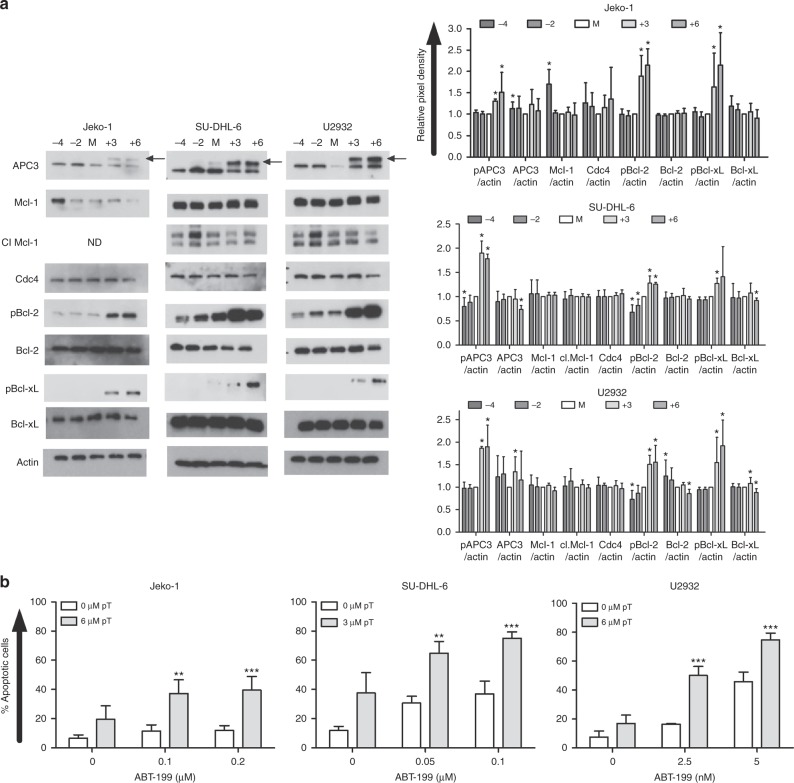
proTAME inactivates the anti-apoptotic proteins Bcl-2 and Bcl-xL and sensitises MCL and DLBCL cells to venetoclax. **a** Effect of proTAME treatment on the expression and phosphorylation of Bcl-2, Bcl-xL and Mcl-1. The expression of Mcl-1, Cdc4, Bcl-2 and Bcl-xL protein and phosphorylation of Bcl-2 and Bcl-xL was determined at different timepoints in the synchronised MCL and DLBCL cell lines after proTAME treatment (3 µM in SU-DHL-6 and 6 µM in Jeko-1 and U2932) using western blot. APC3 and β-actin were used as indicators of mitotic arrest and loading control. Arrows show the phosphorylated APC3. One experiment representative of three is shown (the results shown are from the same experiment as those shown in Fig. 3). Normalisation was performed with Image J and quantification relative to the M condition is shown. Bars represent the mean± SD of three independent experiments. M = mitosis, −4 = 4 h before mitosis, −2 = 2 h before mitosis, +3 = 3 h after mitosis and +6 = 6 h after mitosis, ND = not detected. **b** The anti-lymphoma effect of APC/C targeting in combination with the BH3 mimetic ABT-199. Apoptosis of proTAME (pT) and ABT-199-treated Jeko-1, SU-DHL-6 and U2932 cells was determined after 48 h using Annexin V/7′-AAD staining followed by flow-cytometric analysis. The sum of the percentage of Annexin V and Annexin V/7′-AAD-positive cells are shown. The results shown in each graph are the mean ± SD of four independent experiments. Each combination was compared with both single agents. **p < 0.01 and ***p < 0.001

A prolonged metaphase is known to delay the activation of Cdk1, which can then phosphorylate/inactivate several anti-apoptotic proteins from the Bcl-2 family, thus resulting in the activation of the intrinsic apoptotic pathway.^35^ The effect of proTAME treatment on the expression and phosphorylation of Mcl-1, Bcl-2 and Bcl-xL was evaluated during mitotic arrest by western blot. No clear difference in the total Bcl-2, Bcl-xL and Mcl-1 protein was observed. Nevertheless, consistent with the prolonged activation of Cdk1, an increase in phosphorylation of Bcl-2 and Bcl-xL was observed during the mitotic arrest (Fig. 4a). In contrast, neither a difference was observed in the cleaved Mcl-1 protein levels during a mitotic arrest nor in the Cdc4 (also known as FBW7) protein levels. Again, no changes in phosphorylation of Bcl-2 or Bcl-xL were observed in the untreated synchronised cells (Supplemental Fig. 2). Together, these data suggest that APC/C targeting induces caspase-mediated apoptosis by inactivating the anti-apoptotic protein Bcl-2 and Bcl-xL. Based on this, we next hypothesised that proTAME might sensitise the DLBCL and MCL cell lines to a BH3 mimetic. To investigate this hypothesis, DLBCL and MCL cell lines were treated with proTAME and the selective Bcl-2 inhibitor ABT-199. In all three lymphoma subtypes, an increased percentage of apoptotic cells was observed in the cells treated with proTAME and ABT-199 compared with both single agents (Fig. 4b).

### ProTAME enhances the anti-lymphoma activity of the Cdc20/Cdh1 inhibitor apcin and the standard-of-care agent doxorubicin

**Fig. 5:**
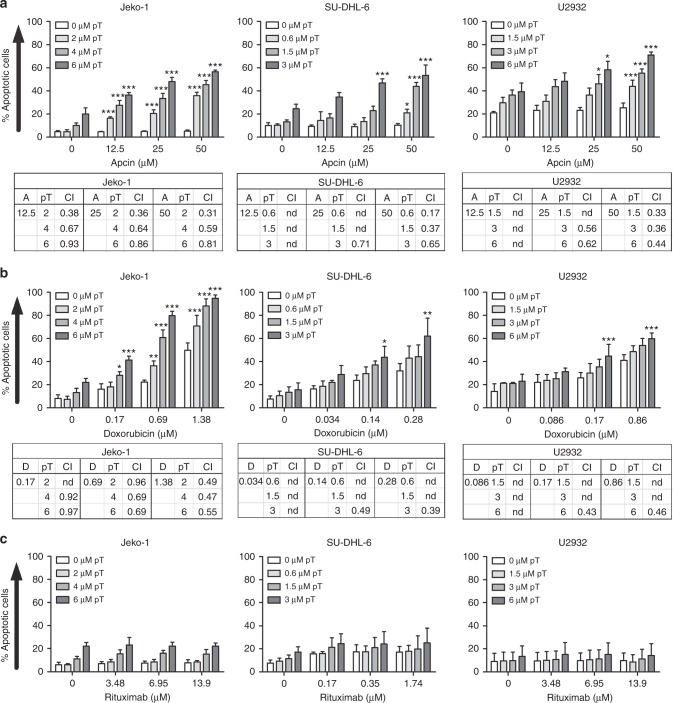
APC/C inhibition enhances the anti-lymphoma activity of the Cdc20/Cdh1 inhibitor apcin and the clinically relevant agent doxorubicin. **a–c** The anti-lymphoma effect of APC/C targeting in combination with apcin, doxorubicin or rituximab. Apoptosis of proTAME (pT) and/or apcin (**a**), doxorubicin (**b**) or rituximab (**c**) treated with Jeko-1, SU-DHL-6 and U2932 cells was determined after 48 h using Annexin V/7′-AAD staining followed by flow-cytometric analysis. The sum of the percentage of Annexin V and Annexin V/7′-AAD-positive cells are shown. The results shown in each graph are the mean ± SD of four independent experiments. *p < 0.05, **p < 0.01 and ***p < 0.001 when compared with both single agents. Combination index (CI) values were calculated for the different drug concentrations by the Chou and Thalalay method using CompuSyn 1.0 software (nd: not determined, CI ≤ 1: synergistic)

A previous study showed that simultaneous disruption of different protein interactions of the APC/C might be a more effective tool for inactivating this complex.^36^ Therefore, proTAME was combined with a Cdc20/Cdh1 inhibitor, namely apcin, which prevents the co-activator–substrate interaction. Different concentrations (IC-10, IC-30 and IC-50) of both agents were used to test the combinatory effect (Supplemental Table 2). The combination of proTAME and apcin significantly increased the effect of both single agents in all human cell lines (Fig. 5a), with the MCL cell line being the most sensitive. Moreover, this combinatory effect was found to be synergistic in all cell lines, with combination indexes well below 1 for the relevant concentrations. Next, to investigate the effect of APC/C inhibition on the activity of clinically relevant drugs, we performed combination studies with the standard-of-care agents doxorubicin and rituximab. ProTAME treatment was found to significantly and synergistically sensitise lymphoma cells of all the B cell NHL subtypes tested to doxorubicin-mediated cell death, especially at higher concentrations (Fig. 5b). Of interest, the MCL cell line Jeko-1 again seemed more sensitive to the combination than the DLBCL cell lines SU-DHL-6 and U2932. In contrast, rituximab showed only very minor cytotoxicity in vitro, and this was not further enhanced when combining rituximab with proTAME (Fig. 5c).

## Discussion

Even with recent improvements in the treatment of lymphoma patients, no difference is detected in the overall survival of MCL patients^37^ and there is still a poor outcome for 30–40% of the DLBCL patients.^3^ The high proliferation rate of these lymphoma cells provides the rationale to use selective antimitotic drugs as a treatment option.^37,38^ In this study, the mitotic exit regulator APC/C is identified as a novel, promising target in ABC–DLBCL, GCB–DLBCL and MCL. From literature, it is known that the activity of the APC/C depends on the interaction with the APC/C co-activators. Changes in the expression/activation of one or both of these co-activators subsequently result in alterations in the APC/C activity.^39^ Thus, to investigate the involvement of the APC/C in DLBCL and MCL disease, we investigated the expression of APC/C co-activators Cdc20 and Cdh1 using publicly available gene expression profiling data from DLBCL and MCL patients. We demonstrated that the APC/C co-activator Cdc20 is significantly overexpressed in DLBCL patients and in aggressive MCL patients compared with indolent MCL. Moreover, high Cdc20 gene expression was correlated with a poor survival outcome in the DLBCL patients. Consistent with our results, high Cdc20 gene expression was repeatedly shown to be associated with poor prognosis in various other cancer types, including breast cancer, multiple myeloma, non-small-cell lung cancer, etc.^15–21^ In contrast, there is still some controversy about the role of Cdh1 in tumorigenesis. Cdh1 is generally considered to be a tumour suppressor.^11^ However, two studies showed that loss of Cdh1 function induces cell death and might be essential for tumour development. Moreover, a recent study observed increased Cdh1 expression in primary MM cells.^27,40,41^ In our study, neither a significant difference in Cdh1 mRNA expression was detected in both DLBCL and MCL patients nor was there a significant correlation between expression levels and survival. Importantly, aggressive B-cell malignancies are a diverse group of lymphomas, including many variants of DLBCL and MCL. There are enormous differences in clinical behaviour and molecular mechanisms, as well as response to treatment. Consequently, identification of new prognostic markers is of utmost importance to achieve tailored therapy.^42^ The Cox analysis in our study revealed that Cdc20 is an independent prognostic factor in DLBCL and Cdc20 protein expression seems to be linked to a poor prognosis in DLBCL patients. Importantly, the prognostic value in DLBCL seems to be irrespective of the molecular subtype and double expressers. Based on these findings, Cdc20 could be a potential new, relevant marker for poor prognosis in DLBCL patients. However, further validation on protein level in a prospective study in larger patient cohorts remains warranted. Together, our data indicate that Cdc20 is involved in DLBCL and MCL pathogenesis, thus supporting the preclinical testing of APC/C targeting in these aggressive B-cell NHLs.

The therapeutic potential of targeting the APC/C, either by blocking the activation of the complex or the co-activators, has been examined in cancer using proTAME,^15,26,27^ withaferin A,^43^ NAHA (a hydroxamic acid derivative)^44^ and apcin.^45^ The small molecule proTAME is the only true/selective APC/C inhibitor known so far.^26^ ProTAME has already been described to cause a prolonged metaphase in cancer cells.^15,26^ Consistent with these observations, synchronised DLBCL and MCL cells released into proTAME treatment were found to accumulate in the G2/M phase. Western blot analysis of these cells demonstrated an increase in cyclin B1 protein levels during this mitotic arrest, while Skp2 levels are not or only slightly affected. The induced G2/M-phase arrest was further investigated, and a significant increase in the percentage of cells in the metaphase was observed. It is known that cells in a prolonged metaphase can either undergo cell death or re-enter the cell cycle to exit mitosis. Cdc20 is believed to be a critical factor in this decision-making. When Cdc20 levels increase during the metaphase, cyclin B1 will be degraded, resulting in Cdk1 inactivation and mitotic exit.^46^ On the other hand, a prolonged activation of the cyclin B1–Cdk1 complex occurs when cells are arrested in the metaphase, resulting in the phosphorylation and inactivation of Bcl-2 and Bcl-xL, and subsequent activation of the intrinsic apoptotic pathway.^35,47,48^ Consistent with the above, the proTAME-induced prolonged metaphase in DLBCL and MCL cells is accompanied by a dose-dependent decrease in viability and increase in caspase-mediated apoptosis. Western blot analysis confirmed the phosphorylation of both Bcl-2 and Bcl-xL. In contrast, Mcl-1 and Cdc4 levels remained mostly unchanged during the mitotic arrest, indicating that the Cdc4–Mcl-1 axis is most likely not involved in proTAME-induced cell death. These findings provide evidence that APC/C targeting using proTAME induces a metaphase arrest in DLBCL and MCL cells, resulting in the accumulation of cyclin B1 and a prolonged activation of Cdk1, followed by phosphorylation of Bcl-2 and Bcl-xL and eventually cell death.

To further ensure that cells will not re-enter the cell cycle after the metaphase arrest induced by proTAME, combination studies were performed. Previously, we and others demonstrated that inactivation of the APC/C is more effective when multiple protein interactions are simultaneously disrupted using proTAME and apcin.^15,36^ ProTAME blocks the interaction between the APC/C and its co-activators, while apcin inhibits the interaction between the co-activators and their substrates.^36^ This combination resulted in a synergistic anti-lymphoma effect in all cell lines. Other studies in myeloma also showed a combinatory effect with the anti-myeloma agents melphalan, vincristine, etoposide and the topoisomerase II inhibitor doxorubicin.^15,27^ Topoisomerase IIα is identified as a Cdh1 substrate and both proTAME and knockdown of Cdh1 significantly enhance the sensitivity of cancers cells to topoisomerase II inhibitors.^49^ Consistent with these findings, proTAME was also found to sensitise all lymphoma cell lines tested to doxorubicin. Combining proTAME with rituximab did not demonstrate any additional effect. This could be explained by the minor effects that rituximab alone had on the different cell lines in vitro. Consistently with our data, others also observed no or only minor effects when using rituximab as monotherapy in DLBCL cell lines.^50^ The anti-lymphoma effect of proTAME combined with a novel agent in clinical development in DLBCL and MCL, namely the selective Bcl-2 inhibitor venetoclax, was also tested. ProTAME strongly and significantly potentiated the anti-lymphoma activity of venetoclax in all cell lines tested, even in the highly sensitive ABC–DLBCL cell line U2932.

To further validate the therapeutic potential of targeting of APC/C in DLBCL and MCL, we acknowledge that in vivo studies are required. Unfortunately, when it comes to the in vivo use of proTAME, there are some concerns. ProTAME has not yet been optimised for in vivo studies, since this prodrug is activated intracellularly by esterases and the active form (TAME) is not cell permeable.^26^ These enzymes can be present in the bloodstream, thus limiting the in vivo use of proTAME. A possible solution could be to pack (pro)TAME in a liposomal vesicle, such as the vesicle used for liposomal doxorubicin in the treatment of aggressive B-cell lymphoma.^51^ Another general concern is that inhibition of the APC/C will most likely affect all dividing cell types. To avoid these off-target effects, liposomal (pro)TAME could be altered, so that it can only be recognised by CD20+B cells. This would give the advantage of specifically targeting the lymphoma cells like rituximab.

In conclusion, this study demonstrates that high Cdc20 expression correlates with a poor prognosis in the aggressive B-cell malignancy DLBCL. We also provide evidence that APC/C targeting in DLBCL and MCL cells results in a prolonged metaphase followed by apoptosis and sensitises the lymphoma cells to the clinically relevant agents doxorubicin and venetoclax. Together, this study suggests that the APC/C is a potential new target in these cancer types, making it a promising target for further preclinical research.

## Supplementary information

Supplementary information

## Data Availability

The datasets supporting the conclusions are included within the article (and its additional files).
