# Supplementary material for: The anaphase-promoting complex/cyclosome: a new promising target in diffuse large B-cell lymphoma and mantle cell lymphoma
Source: Br J Cancer. 2019 May 15;120(12):1137–46. doi: 10.1038/s41416-019-0471-0 (PMC6738099; doi:10.1038/s41416-019-0471-0)
Supplement: Supplementary file 1 — Supplementary information [file 41416_2019_471_MOESM1_ESM.docx]

**SUPPLEMENTARY INFORMATION**

**SUPPLEMENTAL METHODS**

**Primary patient samples**

Cells from lymph node samples were obtained after patients’ written informed consent in accordance with the Declaration of Helsinki and institutional research board approval from Montpellier University hospital. Cells are cultured in IMDM medium (Gibco, Rockville, MD) with 20% FBS with antibiotics-antimicotics (Gibco Penicillin-streptomycin-amphotericin B 100X, #15240-096) at 5x10^5^ cell/mL with 50ng/mL of histidine-tagged CD40L (R&D System, 2706-CL) and 5µg/mL of anti-histidine antibody (R&D System, MAB050). Cells were treated with or without several concentrations of proTAME during 72 hours. Total cell concentration and viability were assessed using trypan blue dye exclusion and the toxicity on DLBCL cells was evaluated using the panel of anti-CD45 V500 (#560777), CD19 PE-Cy7 (#341113), CD3 APC-H7 (#641415), CD10 APC (#332777), CD20 V450 (#655872) (Becton Dickinson), Kappa FITC (Dako, F0434), Lambda PE (Dako, R0437) antibodies before flow cytometry analysis (FACS Canto II cytometer, Becton Dickinson).

**Western blot analysis**

Cells were harvested, lysed and western blot was performed as previously described [1]. Antibodies were used against APC3 (#12530), Cyclin B1 (# 4138), Skp2 (# 4313), pBcl-2 (#2827), Bcl-xL (#2764), Mcl-1 (# 5453), Cdc20 (# 4823) and β-actin (#4967) (all from Cell Signaling, Leiden, the Netherlands), Bcl-2 (sc-492), pBcl-XL (sc-101644), Cdc4 (sc-293423) and Cdh1 (sc-19398) (all from Santa Cruz, Heidelberg, Germany). The pixel density of proteins was quantified by ImageJ.

**Quantitative real-time PCR**

Total RNA was extracted using the Nucleospin RNA plus kit (Macherey-Nagel, Düren, Germany) and reverse transcription was performed using the Verso cDNA synthesis kit (ThermoFisher Scientific, Gent, Belgium), both according to manufacturer’s instructions. Quantitative real-time PCR was performed as previously described [1]. Primers for Cdc20 and Cdh1 were purchased from IDT (Leuven, Belgium and primers for GAPDH were purchased from Qiagen (Venlo, The Netherlands). Primer sequences were as followed (5’-3’): Cdc20 forward: CTG GAT CAA AGA GGG CAA CTA; reverse: GGC AGA GTG ACT GGT CAT ATT and Cdh1 forward: GGA TGT CTG CTC TGG ACT TAT C; reverse: AAG ACG TGG CAT CTG TTG T.

**Cell viability assay**

The CellTiter-Glo Luminiscent Viability assay (Promega, Leiden, The Netherlands) was used to measure cell viability according to manufacturer’s instructions.

**Apoptosis assay**

Apoptosis was quantified using an Annexin V /7’-AAD staining (BD Biosciences, Franklin Lakes, USA) and active caspase 3 staining (BD Biosciences), followed by flow cytometric analysis.

**Cell synchronization**

Cells were synchronized using a double thymidine block. Thymidine (Sigma-Aldrich) was added at a concentration of 2mM for 18 hours. After 9 hours of culturing in fresh medium, cells underwent a second thymidine block for 15 hours. When released into fresh medium, cells were synchronized at the G1/S border and released into S phase.

**Cell cycle analysis**

Propidium iodide (PI) staining was used to analyze cell cycle distribution. Cells were incubated with a PI solution containing 1mg/ml sodium nitrate (Merck KGaA, Darmstadt, Germany), 0.1% Triton-X (Merck), 100µg/ml RNase A (Boehringer, Ingelheim, Germany) and 50µg/ ml PI (Sigma-Aldrich). Cells were analyzed by flow cytometry.

**Assessment of the amount of cells in metaphase**

Cytospins (100 000 cells/slide) were stained with May-Grünwald Giemsa (Merck KGaA). Based on morphology, cells in metaphase were counted under a light microscope. Three fields of 100 cells were counted for each cytospin.

**Cell count**

Cells in culture were stained using trypan blue solution (Sigma-Aldrich) and viable cells were counted under a light microscope.

**B cell isolation from blood samples**

Peripheral mononuclear cells were obtained from whole blood from healthy donors and separated by Ficoll Hypaque (Nycomed, Lucron Bioproducts, De Pinte, Belgium) gradient centrifugation. B cells were isolated using B cell isolation kit (Miltenyi Biotec, Bergisch Gladbach, Germany) according to manufacturer’s instructions.

**SUPPLEMANTAL FIGURES**

**Supplemental Figure 1: Pharmacological inhibition of APC/C in lymphoma cell lines.**

**(A) Cdc20 and Cdh1 expression in a large panel of MCL and DLBCL cell lines.** Cdc20 and Cdh1 gene expression levels of B cell samples (n=33) and different DLBCL (n=13) and MCL cell lines (n=5) was obtained from the GEP datasets GSE56315 and GSE36133. Mean expression ± SD is shown in red. **(B-C) Basal Cdc20 and Cdh1 expression in a selected panel of MCL and DLBCL cell lines.** Basal Cdc20 and Cdh1 mRNA (B) and protein (C) levels were determined in isolated B cells, MCL (Mino, Jeko-1 and Rec-1) and DLBCL cell lines (OCI-Ly1, OCI-Ly7, SU-DHL-6, RI-1 and U2932) using quantitative real-time PCR and western blot respectively. Mean ± SD of 3 independent experiments is shown for quantitative real-time PCR and one experiment representative of 3 is shown for western blot. **(D) Effect of proTAME treatment on cell count.** MCL and DLBCL cell lines were treated for 24 hours with proTAME (3, 6 and 12 µM) and the effect on cell count was determined using trypan blue staining. **(E-H) Effect of proTAME treatment after 48 hours.** MCL and DLBCL cell lines were treated for 48 hours with proTAME (3, 6 and 12 µM) and the effect on cell count (E), cell viability (F), apoptosis (G) and active caspase 3 (H) was determined. Results shown are mean ± SD of 3 independent experiments.

**Supplemental Figure 2:** **Effect of synchronization on APC/C substrates and Bcl-2 protein family.**

The expression of cyclin B1, Skp2, Mcl-1, Bcl-2 and Bcl-xL protein and phosphorylation of Bcl-2 and Bcl-xL was determined at different timepoints in the synchronized MCL and DLBCL cell lines using western blot. APC3 and β-actin were used respectively as indicator of mitotic arrest and loading control. One experiment representative of 3 is shown. Normalization was performed with Image J and quantification relative to the M condition is shown. Bars represent the mean ± SD of 3 independent experiments. M = mitosis, -4 = 4 hours before mitosis, -2 = 2 hours before mitosis, +3 = 3 hours after mitosis, +6 = 6 hours after mitosis, ND = not detected.

**Supplemental Figure 3: APC/C targeting using proTAME results in a prolonged metaphase.**

May-Grünwald Giemsa staining of synchronized Jeko-1, SU-DHL-6 and U2932 cells released into proTAME (3µM for SU-DHL-6 and 6µM for Jeko-1 and U2932) at different time points. A 400x magnification is shown. M = mitosis,-2 = 2 hours before mitosis, +3 = 3 hours after mitosis, +6 = 6 hours after mitosis.

**REFERENCES**

1. De Bruyne E, Bos JT, Schuit F, Van Valckenborgh E, Menu E, Thorrez L, et al. IGF-1 suppresses Bim expression in multiple myeloma via epigenetic and posttranslational mechanisms. Blood. 2010;115(12):2430–40.


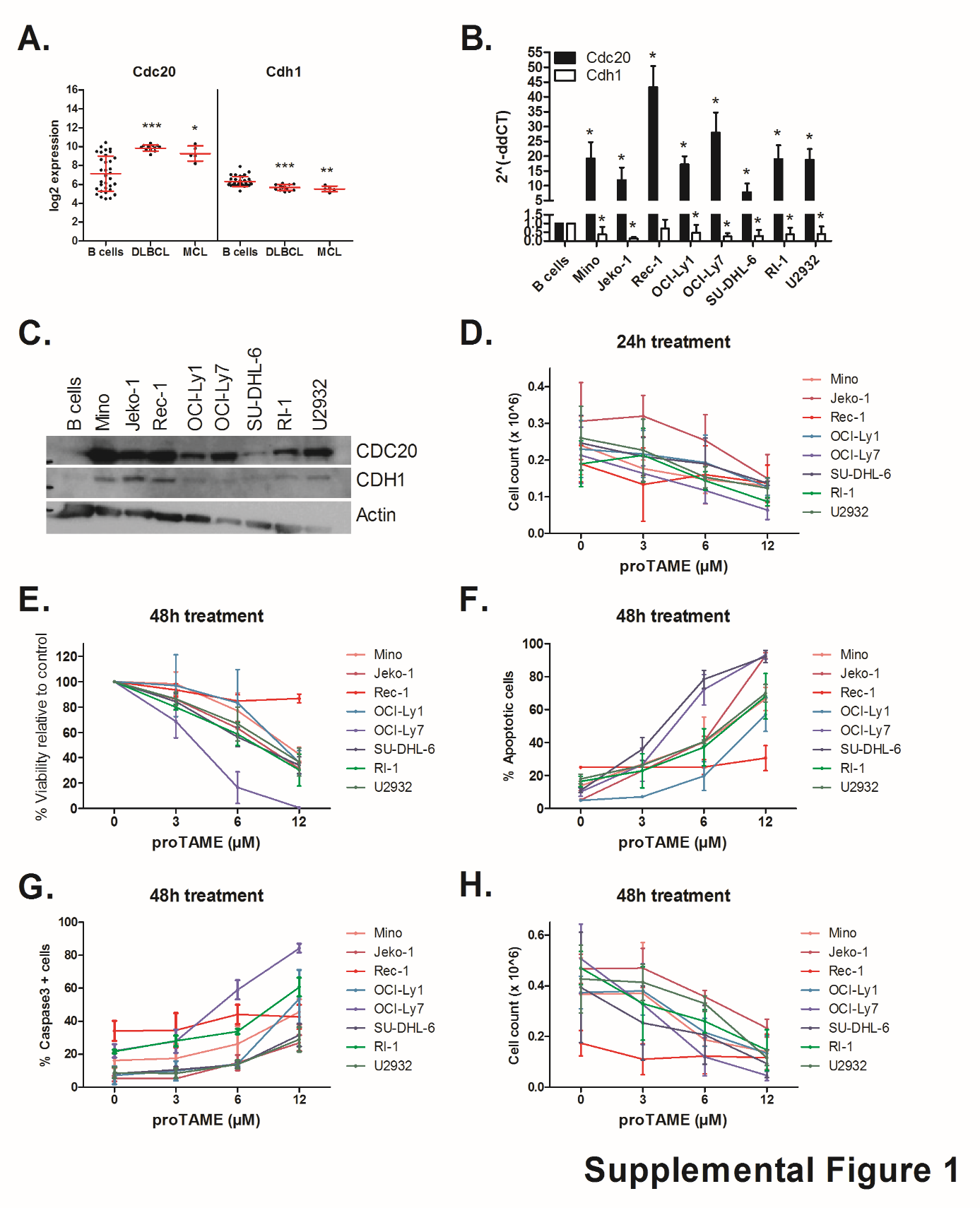


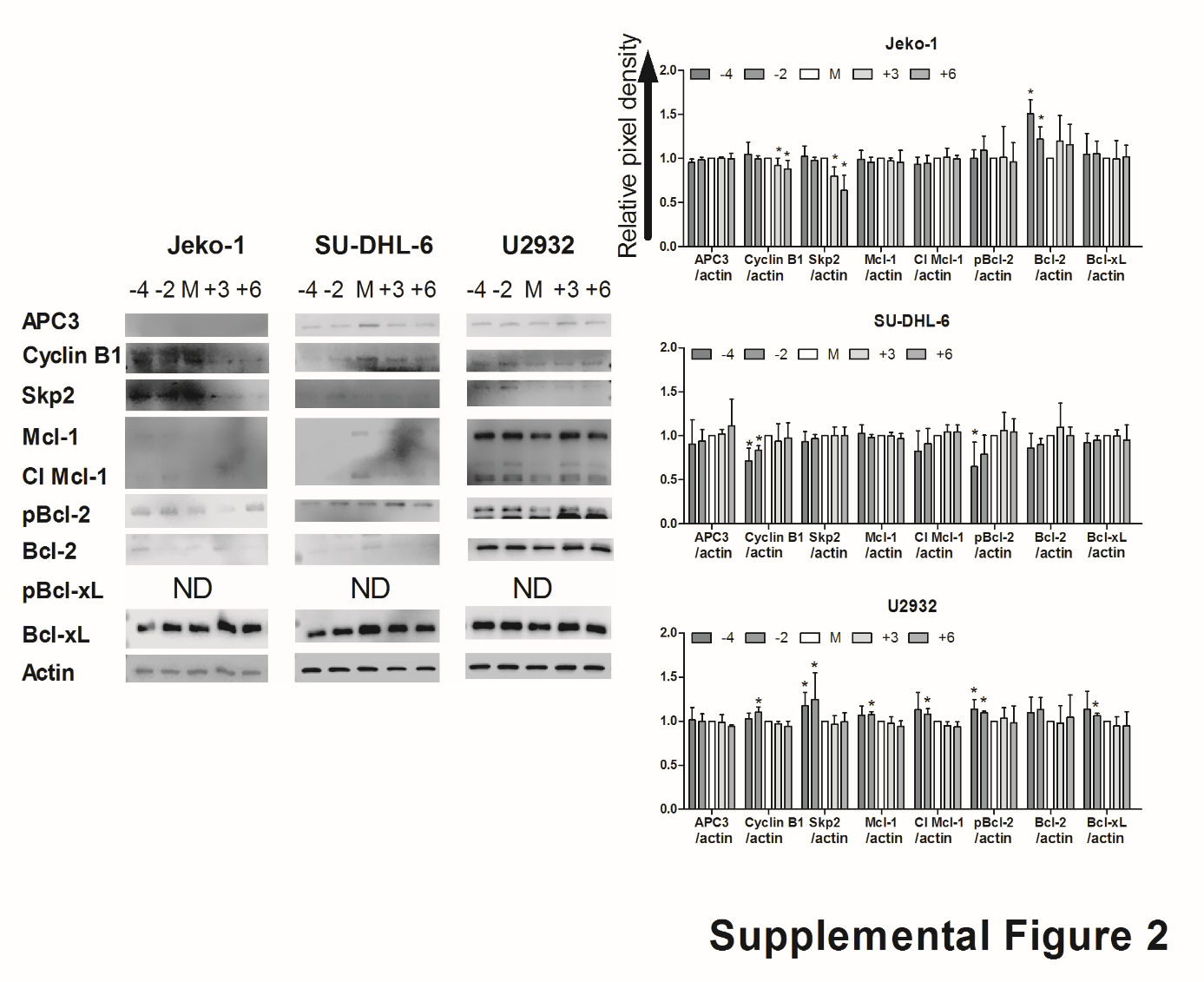


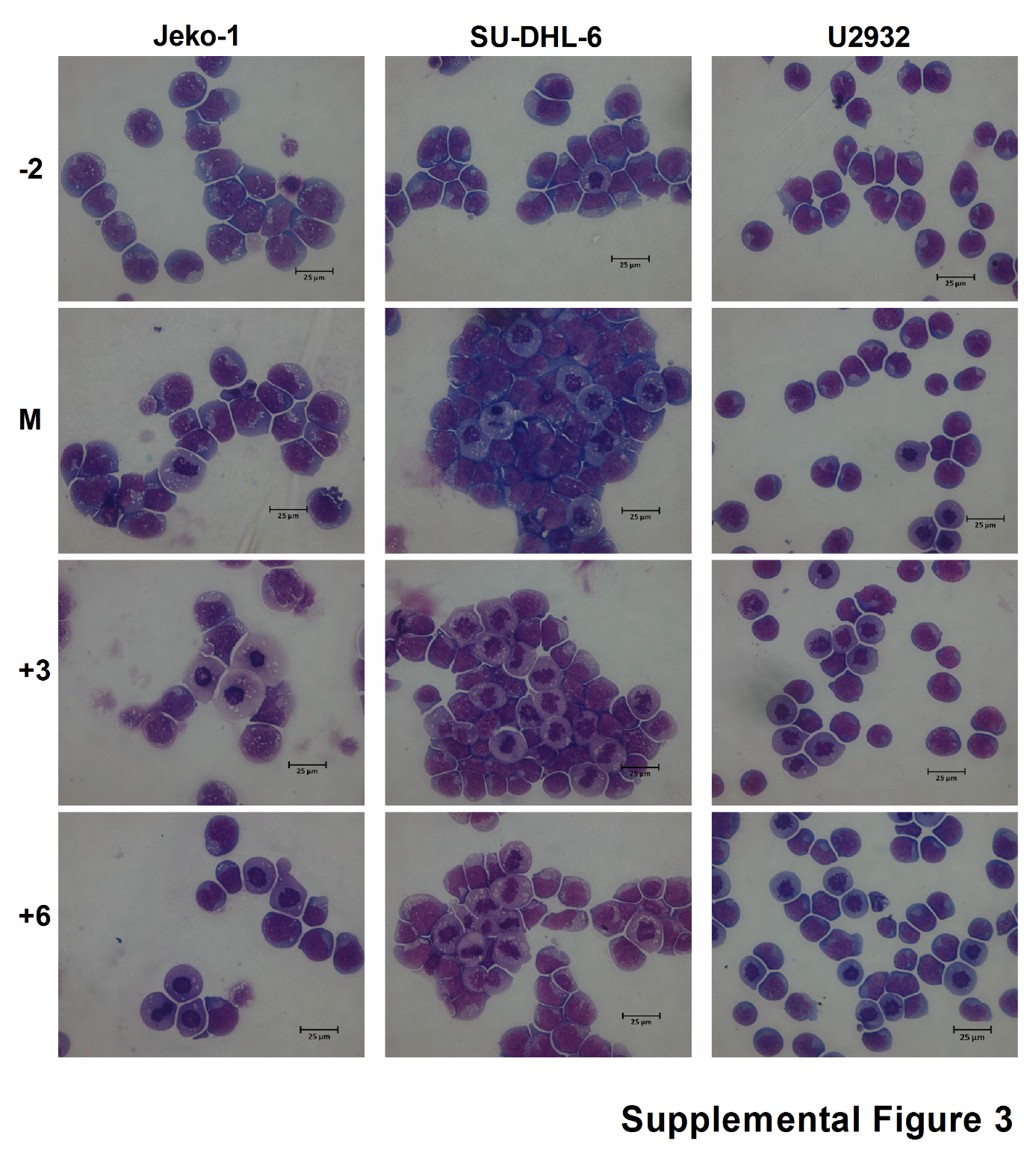


**SUPPLEMENTAL TABLE 1**

| Disease | Patient | Gender | Age | Subtype | Staging | Patient status |
| --- | --- | --- | --- | --- | --- | --- |
| MCL | #1 | M | 42 | Indolent |  | Progression |
|  | #2 | M | 74 | Indolent |  | Progression |
|  | #3 | F | 68 | NOS | MIPI: 4 Ann Arbor Stage: IV | Relapse |
|  | #4 | M | 73 |  |  | Diagnosis |
| DLBCL | #1 | M | 71 | Non-GCB | IPI: 4 Ann Arbor Stage: III | Diagnosis |
|  | #2 | M | 78 | Non-GCB | IPI: 5 Ann Arbor Stage: IV | Diagnosis |
|  | #3 | M | 81 | Non-GCB | Ann Arbor Stage: III | Diagnosis |

*NOS: not otherwise specified and (M)IPI: (Mantle cell lymphoma) International Prognostic Index*

**Supplemental Table 1: Patient characteristics**

**SUPPLEMENTAL TABLE 2**

| **A.** | **Jeko-1** | | | **SU-DHL-6** | | | **U2932** | | |
| --- | --- | --- | --- | --- | --- | --- | --- | --- | --- |
| Reagent | IC-10 | IC-30 | IC-50 | IC-10 | IC-30 | IC-50 | IC-10 | IC-30 | IC-50 |
| proTAME (µM) | 2 | 4 | 6 | 0.6 | 1.5 | 3 | 1.5 | 3 | 6 |
| Doxorubicin (µM) | 0.17 | 0.69 | 1.38 | 0.034 | 0.14 | 0.28 | 0.086 | 0.17 | 0.86 |
| Rituximab (µM) | 3.48 | 6.95 | 13 .9 | 0.17 | 0.35 | 1.74 | 3.48 | 6.95 | 13.9 |

**Supplemental Table 2:** The IC-10, IC-30 and IC-50 values for proTAME, doxorubicin and rituximab for the different cell lines after 48 hours of treatment.
